# Anticandidal Activities by *Lactobacillus* Species: An Update on Mechanisms of Action

**DOI:** 10.3389/froh.2021.689382

**Published:** 2021-07-16

**Authors:** Roberto Vazquez-Munoz, Anna Dongari-Bagtzoglou

**Affiliations:** Department of Oral Health and Diagnostic Sciences, University of Connecticut Health Center, Farmington, CT, United States

**Keywords:** *Lactobacillus*, *Candida*, dysbiosis, microbiome, candidiasis, probiotics, microbiota

## Abstract

Lactobacilli are among the most studied bacteria in the microbiome of the orodigestive and genitourinary tracts. As probiotics, lactobacilli may provide various benefits to the host. These benefits include regulating the composition of the resident microbiota, preventing – or even potentially reverting- a dysbiotic state. *Candida albicans* is an opportunistic pathogen that can influence and be influenced by other members of the mucosal microbiota and, under immune-compromising conditions, can cause disease. *Lactobacillus* and *Candida* species can colonize the same mucosal sites; however, certain *Lactobacillus* species display antifungal activities that can contribute to low *Candida* burdens and prevent fungal infection. Lactobacilli can produce metabolites with direct anticandidal function or enhance the host defense mechanisms against fungi. Most of the *Lactobacillus* spp. anticandidal mechanisms of action remain underexplored. This work aims to comprehensively review and provide an update on the current knowledge regarding these anticandidal mechanisms.

## Introduction

According to a Human Microbiome Project definition –initially proposed by Lederberg and McCray in 2001 [1], the term microbiota comprises the communities of symbiotic and pathogenic microbes hosted by multicellular eukaryotic organisms –including humans- [2]. An individual may host trillions of these microorganisms –mostly bacteria- with whom they interact physically, chemically, and biologically. The interactions between the host and its associated microbiota as a whole are complex, dynamic, and mostly unknown. Understanding what a “healthy microbiome” is, its composition, and its impact on host health has been a challenge for decades [3]; however, the significant role indigenous microbiota play on the health of the host is well-established [4, 5]. Health-associated microbiota and their metabolic products may play a role in treating or preventing infections. For example, probiotics –live microorganisms-, paraprobiotics –non-viable microbial cells-, and post-biotics –soluble factors produced by bacteria are currently used in food products to promote health.

Around 98% of the microbial constituents of the oral and gastrointestinal (GI) tract of healthy humans fall within four predominant bacterial phyla: Firmicutes, Bacteroidetes, Actinobacteria, and Proteobacteria [3, 6]. The role of bacteria in maintaining oral health has been studied extensively; in contrast, fungal microbiota –comprising members of the mycobiome– remain mostly unexplored [7, 8]. Microbial composition and diversity in the oral cavity and GI tract have also been studied extensively. Environmental filtering and competitive exclusion shape microbial composition, favoring the presence of taxa distantly related to each other, resulting in a greater diversity –at the species level- among healthy hosts. In contrast, closely related taxa are favorably selected within an individual host [9]. Additional factors that shape microbiota diversity are host genetic variation, age, diet, and the ecological local microbial environment, among others [5, 10, 11]. Although microbiota may change over time, any major disruption that significantly alters the microbiota can lead to severe health consequences [12, 13].

Disruption of the health-associated microbiota contributing to infectious and non-communicable systemic diseases is known as dysbiosis. A dysbiotic state may result as a consequence of medical therapies such as antibiotics [14], cancer chemotherapy [15], metabolic disorders such as obesity [16], or infection with pathogenic fungi [17], bacteria [18], or viruses [19]. A dysbiotic state may aggravate inflammatory disorders such as inflammatory bowel disease, asthma, neurodegenerative diseases, and immunopathology [12, 20, 21]. Dysbiosis is also associated with the pathogenesis of metabolic disorders, such as obesity, insulin resistance, and dyslipidemia [22]. Moreover, under a dysbiotic scenario, some pathobionts (commensals that can turn into opportunistic pathogens) may form synergistic interactions with each other, leading to severe infections. Recently, Bertolini et al. showed that oral bacterial pathobionts facilitate fungal infection by increasing the expression of virulence genes in *C. albicans* or disrupting the mucosal barrier *via* proteolytic enzymes [23]. Hong et al. suggested oral microbiota disruption is associated with oral mucositis, exacerbating epithelial injury in cancer patients receiving cytotoxic chemotherapy [15].

In this paper, we provide a comprehensive review of the current understanding of the role of lactobacilli in maintaining mucosal homeostasis, with particular emphasis on their interactions with *Candida* species and mucosal candidiasis. All PubMed available information was analyzed and discussed under their respective thematic sections. We particularly focused on studies that described a mechanism of action, including *in vitro, ex vivo, in vivo*, and clinical trials.

## *Candida* and *Lactobacillus* Influence on Host Health and Resident Mucosal Microbiota

### Candidiasis and Dysbiosis

*Candida albicans* is a polymorphic yeast from the *Candidaceae* family. This yeast is a part of the skin, vaginal, oral, and gastrointestinal microbiota in most healthy humans [24]. *Candida albicans* has been traditionally defined as an opportunistic pathogen, and more recently as a pathobiont. *Candida* causes damage to the host under certain conditions, resulting in local (skin, mucosa) or systemic (blood, urinary, nervous system) candidiasis. The ability of *C. albicans* to transition between different morphological forms is a crucial factor for mucosal virulence [25, 26]. *Candida albicans* is the most common opportunistic fungal pathogen and the leading cause of death in fungal diseases [27]. Additionally, drug-resistant *C. albicans* strains and other *Candida* species such as *C. auris, C. glabrata*, and *C. parapsilosis*, are listed by the CDC as a serious threat to human health, as they are challenging to treat. Moreover, many non-*albicans* species are challenging to detect and identify [28, 29]. *Candida* species can form biofilms both on tissues and abiotic surfaces, increasing their survival under harsh environments and their resistance to antifungal drugs and disinfectants [30, 31].

Several reports show that mucosal candidiasis occurs at higher rates in immunocompromised patients [32, 33] as a consequence of the interplay between a defective innate or adaptive immunity and fungal virulence factors [18, 34, 35]. Candidiasis may influence and be influenced by members of the mucosal microbiome [36]. For example, a synergistic interaction between *C. albicans* and mitis group streptococci which was first identified experimentally [13, 37], may have clinical implications on Autosomal-Dominant Hyper IgE Syndrome patients, who are susceptible to this oral infection [21]. An example of Candida influencing members of the bacterial microbiome is the interaction between *C. albicans* and *Staphylococcus aureus*, which promotes bacterial colonization and systemic dissemination [38]. Additionally, *Candida* may alter the reestablishment of resident bacterial communities after broad-spectrum antibiotic treatment. *Candida albicans* alters the proportion and genus diversity within the main bacterial families (Lachnospiraceae, Ruminococcaceae, Rikenellaceae, and Porphyromonadaceae) in the mouse gut, favoring the overgrowth of *Enterococcus* and *Streptococcus* species while preventing the reestablishment of *Lactobacillus* and *Oscillibacter* [39, 40]. This disturbance in post-antibiotic communities may contribute to fungal infection since both enterococci and streptococci have been implicated in fungal pathogenic synergy [13, 17]. On the other hand, several bacterial members of the healthy microbiota display direct anticandidal properties or promote host anticandidal responses. Such bacterial species with broad influence on bacterial and fungal members of the microbiome are *Lactobacillus* spp., which may prevent dysbiosis and associated diseases, as described in the following sections.

### Basic Physiology and Current Taxonomy of the Genus *Lactobacillus*

*Lactobacillus* spp. belong to the family *Lactobacillaceae*, a taxon of Gram-positive, rod-shaped bacteria whose main end metabolic product is lactic acid. Lactobacilli are facultative-anaerobic, non-motile, catalase-negative, psychrophilic microorganisms that do not form spores. Since their discovery more than 100 years ago, research regarding their evolution, natural history, and organization of the groups within the genus is under constant change. A decade ago, Singh et al. described the challenges regarding establishing the taxonomy within the genus *Lactobacillus* due to differences in the 16S rRNA gene, resulting in taxonomic uncertainty even in the four main groups –*L. acidophilus, L. casei, L. plantarum*, and *L. delbrueckii*- and their members [41]. Recently, an update by Duar et al. examined the phylogenetic, genomic, and metabolic information available to resolve the natural history of the genus, resulting in a new organization of the groups within the genus *Lactobacillus*, such as the *L. reuteri* group and moving the *L. acidophilus* group within the *L. delbrueckii* group [42]. Zheng et al. examined the physiology, ecology, core phylogeny, and sequence comparisons of 16S rRNA gene and clade-specific genes. This study resulted in the organization of the genus *Lactobacillus* into 26 phylogenetic groups, including creating new genera, which remain closely related to the genus *Lactobacillus* [43]. The classification by Zheng et al. has been adopted in the List of Prokaryotic names with Standing in Nomenclature (LPSN), resulting in significant changes, as follows [43, 44]: *Lactobacillus casei* group is now the genus *Lacticaseibacillus* (including *L. casei, L. paracasei*, and *L. rhamnosus*), *Lactobacillus plantarum* is now the genus *Lactiplantibacillus* (including *L. plantarum, L. paraplantarum*, and *L. pentosus* species.), and *L. reuteri* group is now the genus *Limosilactobacillus* (including species such as *L. reuteri*, and *L. vaginalis*). *Lactobacillus delbrueckii* group remains within the genus *Lactobacillus* (including species such as *L. acidophilus, L. crispatus, L. gasseri, L. jensenii, L. johnsonii*, and the *L. delbrueckii* subspecies such as *L. delbrueckii* ssp. bulgaricus and *L. delbrueckii* ssp. lactis). Due to the difficulty of differentiating between closely related *Lactobacillus* species (even with molecular techniques) and the proximity of lactobacilli to other genera, the term “*Lactobacillus* Genus Complex” describe lactobacilli and closely related taxa, such as a newly created genus from former *Lactobacillus* species, *Leuconostococcaceae*, and *Pediococcus* [42, 43, 45]. For this review, the species which were formerly members of the genus *Lactobacillus* and now belong to closely-related new genera –i.e., *Lacticaseibacillus* and *Lactiplantibacillus-* will be considered as members of the genus *Lactobacillus*. As the taxonomy of the *Lactobacillus* genus complex becomes more accurate, the identification of common biological traits within species of the same group –i.e., metabolite production, pili-like structures, etc.- is expanded, leading to a better understanding of the interactions with other members of the microbiota and the host.

### Role of Lactobacilli in the Protection of Mucosal Barriers

Lactobacilli colonize diverse plants and animals, including humans [42], and have co-evolved with their hosts, becoming a stable member of the resident microbiota [46, 47]. Some lactobacilli display a pilus-like structure [48], which may be associated with probiotic properties. The pilus-associated proteins have only been studied in bacteria from the *L. casei* group [49–51], mainly in *L. rhamnosus* GG (LGG) [52]. This pilus-like structure facilitates binding to the mucus associated with mucosal epithelia [53] and plays a role in immunomodulation by reducing the expression of pro-inflammatory molecules (i.e., IL-6, TLR3) [54]. A recent review described the role of *L. rhamnosus* GG (LGG) in protecting the mucosal barrier, the effect of their metabolites on host cells, and their role on type 1 immune-responsiveness [55]. Like *C. albicans*, lactobacilli colonize mucosal tissues of the oral, gastrointestinal, and genitourinary tracts. As lactobacilli have been studied for decades, there is mounting evidence showing that they regulate metabolic processes of the host and other microorganisms and influence the host microbiota composition [56–58]. The FDA and the NIH ascertain that lactobacilli are GRAS (Generally Recognized As Safe) microorganisms associated with health and nutritional benefits [59, 60]. Lactobacilli are currently used in various food products and in treating gastrointestinal (GI) and non-GI medical conditions [61]. Among the different clinically relevant properties found in lactobacilli, their broad antimicrobial activity has attracted particular interest.

### Effects of *Lactobacillus*-Based Therapies on Human Disease

Recent multiple clinical studies, including double-blind, randomized placebo-controlled studies, confirm lactobacilli used as probiotics reduce infections in patients, with no serious adverse events [62–64]. Skrzydło-Radomańska et al. reported that adult Irritable Bowel Syndrome (IBS) patients treated with a probiotic combination –from the species *Lactobacillus* and *Bifidobacterium*- may show reduced symptoms throughout 8-week treatment [65]. Yoon et al. showed that consumption of fermented milk containing a combination of *L. paracasei* and the plant *Glycyrrhiza glabra* reduced *Helicobacter pylori* numbers, inflammation, and gastrointestinal symptoms –indigestion, diarrhea, and abdominal pain- in infected patients [66]. Acute uncomplicated diverticulitis patients treated with a combination of *L. reuteri* and an antibiotic therapy showed reduced abdominal pain and inflammatory markers. Treated patients spent fewer hours of hospitalization than the placebo [67]. These studies also conclude that no serious adverse events have been observed in patients treated with lactobacilli.

Moreover, recent meta-analyses and systematic reviews conclude that evidence regarding the effectiveness of lactobacilli and other probiotics in combating infectious diseases *in vivo* is robust [68–74]. Several meta-analyses have shown that certain *Lactobacillus* species effectively reduce the burden of bacterial infections in patients, such as those from *Helicobacter pylori* [62, 75] or *Clostridium difficile* [63]. Ng et al. concluded that current data demonstrate that lactobacilli effectively combat urinary tract infections in females [64]. A recent meta-analysis by Szajewska et al. concluded that LGG reduces the duration of diarrhea and hospitalization in acute gastroenteritis pediatric patients [68]. Another meta-analysis by Gao et al. suggested that *Lactobacillus* spp can be a beneficial adjunct to non-surgical treatment of dental or peri-implant-associated inflammation initiated by bacterial biofilms [69]. Similarly, a systematic review suggested that lactobacilli –primarily *L. rhamnosus* GR-1 and *L. reuteri*- are beneficial in preventing and even treating recurrent urogenital infections, such as bacterial vaginosis [70]. Overall, despite the fact that some studies report no effect, comprehensive pooled data support the effectiveness of *Lactobacillus*-based probiotic therapies in bacterial infections.

There has been significant interest in using lactobacilli to combat candida infections. Earlier systematic reviews of older reports suggested that data regarding the antifungal activity of lactobacilli were inconsistent and inconclusive [70, 71], proposing that more research was needed due to the significance of the topic. As more studies have been conducted, there is more evidence supporting the anticandidal properties of lactobacilli. A clinical study showed that consumption of milk supplemented with *L. rhamnosus* reduced the severity of *Candida*-associated denture stomatitis in institutionalized elders by decreasing the prevalence of *C. albicans* [72]. Another clinical study showed that patients with the Familial Mediterranean Fever genetic disease displayed lower *C. albicans* burdens after treatment with *Lactobacillus acidophilus* [73]. A clinical study on very-low-birth-weight (VLBW) infants showed that *Candida* colonization was reduced by a prophylactic *L. reuteri* supplementation, which was as effective as the antifungal nystatin treatment and safer, as VLBW patients showed lower feeding intolerance and hospitalization time [74]. Women diagnosed with vulvovaginal candidiasis showed reduced vaginal discomfort and healthier vaginal pH after treatment with conventional treatment supplemented with *L. plantarum* [76].

Thus, there is a wide range of potential clinical applications of *Lactobacillus*-based therapies, even though currently their incorporation in clinical practice is at best limited. As lactobacilli and their metabolites display broad antimicrobial activity extending to bacteria and fungi, understanding their mechanisms of action is essential. Yet, most of the research thus far focused on antibacterial functions, leaving the antifungal mechanisms understudied. In the following sections, we review current knowledge regarding (1) the anticandidal mode of action of lactobacilli-derived metabolites; and (2) the interactions within the *Lactobacillus*-*Candida*-Host framework that prevent *Candida* infections.

## Anticandidal Properties of Lactobacilli

*Candida* spp. infections are an increasing concern as drug-resistant strains are rising while the effectiveness of conventional antifungal therapies is diminishing. Consequently, innovative alternatives for preventing and treating *Candida*-related diseases have been explored, such as drug-repurposing [77], development of new molecules [78], nanotechnology [31, 79], and using other microorganisms as probiotics [80]. *Lactobacillus* species display promising anticandidal activity, yet knowledge on their modes of action is still scarce. The current knowledge regarding these anticandidal mechanisms is comprehensively reviewed and critically appraised in this review. We categorize the lactobacilli-mediated anti-*Candida* activity into direct and indirect. Direct anticandidal activity is primarily caused by bacterial metabolites that kill or inhibit the growth of yeast cells or prevent attachment, dimorphic transition, and biofilm formation. Indirect activity mechanisms rely on *Lactobacillus*-host interactions, as they involve modulation of immune processes or epithelial responses that prevent *Candida* growth on mucosal surfaces and protect the integrity of the mucosal barrier.

*Candida* and lactobacilli can colonize the same host sites, including the oral, pharyngeal, intestinal, and vaginal mucosae, since they are capable of adapting to a wide variety of environments, likely due to their genomic plasticity [81]. However, mucosal colonization in higher abundance by these organisms may be promoted by distinct nutrient profiles, oxygen tension, temperature, pH, and binding to site-specific mucins or epithelial receptors in each site. For example, lactobacilli are the dominant taxa in the healthy vaginal mucosa, characterized by acidic pH and low oxygen content, since they are aciduric facultative anaerobic organisms. In contrast, *C. albicans* colonize this site asymptomatically in low numbers, only in about 20% of healthy women [82]. In this site, alkalization may lead to dysbiosis, reducing *Lactobacillus* abundance and overgrowth of *Candida*, resulting in candidiasis [83]. In addition to acidic pH and low oxygen tension, a key ecological determinant for the sustained colonization of lactobacilli is retentive areas such as crypts and rugal folds in the stomach and carious lesions in the oral cavity [84]. Although *C. albicans* has a broad tolerance to pH and can grow well as planktonic yeast cells, their ability to transition to the hyphal phase and adhere to mucosal sites is impaired in acidic pH [85]. Thus, pH acidification by *Lactobacillus*-produced lactate limits the ability of fungi to cause disease in such mucosal sites. Below we provide a comprehensive analysis of a wide variety of mechanisms employed by lactobacilli which can limit *Candida* overgrowth on mucosal surfaces.

### Direct Antifungal Activity

**Figure 1:**
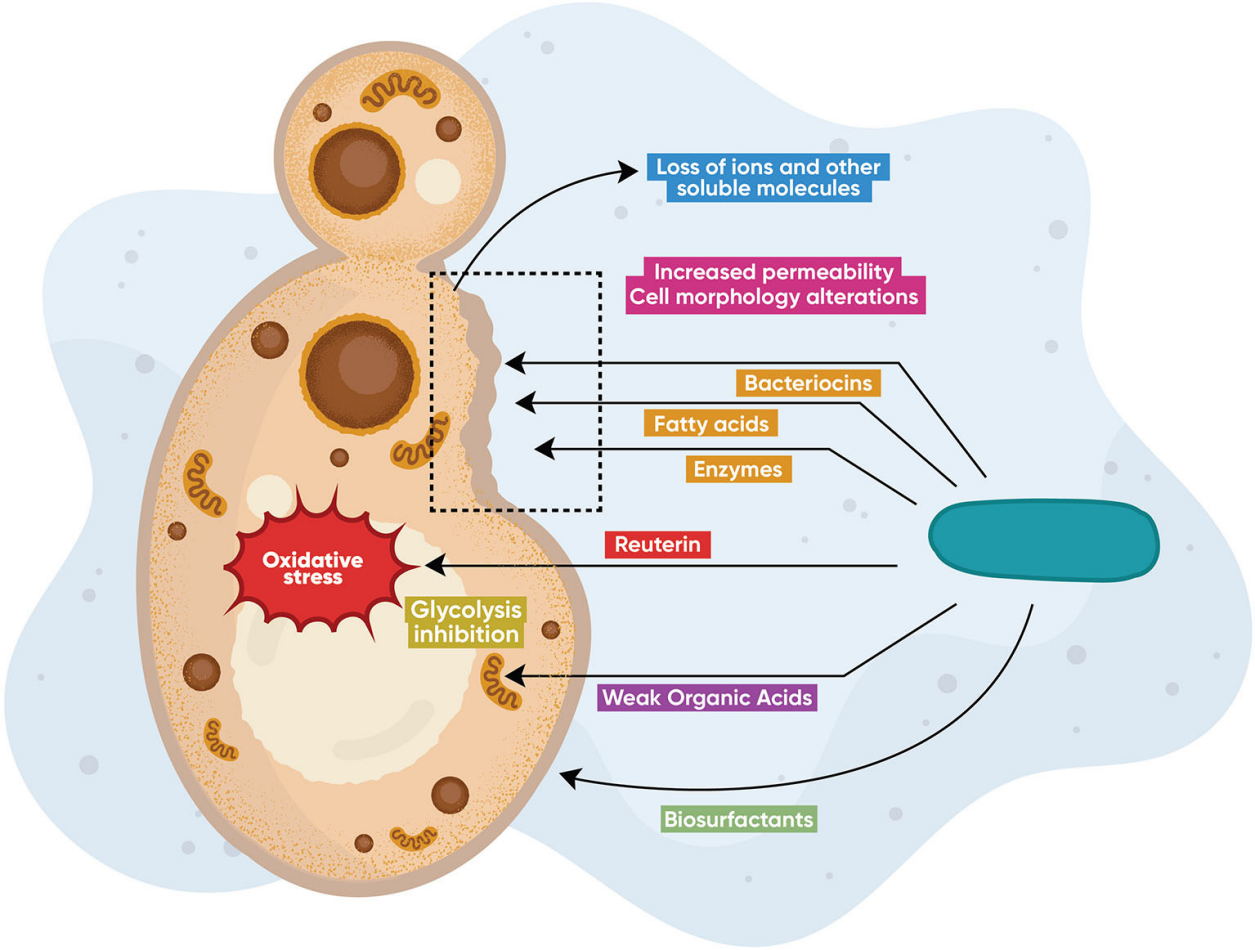
Direct antifungal modes of action. *Lactobacillus* species produce diverse metabolites including bacteriocins, enzymes (chitinases), hydrogen peroxide, fatty and other organic acids, as well as proteinaceous molecules (reuterin), that exert direct anticandidal activity *via* different mechanisms of action. Some metabolites alter the physiology of the fungus by inducing oxidative stress or by ATP depletion leading to cytotoxicity or growth inhibition. Other metabolites compromise the structural integrity of the fungal cell leading to alterations in cell morphology, membrane permeability, and death, while biosurfactants prevent adhesion to mucosal surfaces.

Lactobacilli produce a variety of active compounds (primary and secondary metabolites) that exhibit broad antimicrobial activity (Figure 1). A number of these metabolites are also effective against *C. albicans*. The most studied metabolites are bacteriocins [86] and bacteriocin-like peptides [87], cyclic dipeptides [88], proteinaceous compounds [89], enzymes [90], fatty acids [91], biosurfactants [92, 93], and other organic compounds such as reuterin [94], 3-Phenyllactatic acid [88], and LBP102, derived from *L. plantarum* NTU 102 [95]. Lactobacilli also produce inorganic compounds, such as hydrogen peroxide [96], leading to oxidative stress and genotoxicity in higher amounts. These compounds display broad antimicrobial activity against bacteria and fungi [97–100]. Current knowledge on specific *Lactobacillus*-produced metabolites and their respective anticandidal mechanisms is summarized in Table 1. In Figure 1, the main anticandidal mechanisms of action exerted by *Lactobacillus* products are graphically shown. Several species produce diverse metabolites that alter the physiology of the fungus by inducing oxidative stress and ATP depletion leading to cytotoxicity or growth inhibition. Other metabolites compromise the structural integrity of the fungal cell leading to alterations in cell morphology, membrane permeability, and death, while biosurfactants prevent adhesion to mucosal surfaces, as seen in Figure 1.

**Table 1:** Known anticandidal mechanisms of action from lactobacilli metabolites.

***Lactobacillus* species**	**Anticandidal metabolites**	**Mechanisms of action**	**References**
*L. plantarum*	Bacteriocins.	**Plantaricin** (class I) (1) forms pores in the cell membrane that increase its permeability, (2) alter the yeast cell morphology.	[101]
*L. acidophilus L. plantarum*	Weak organic acids: acetic, lactic, benzoic, and sorbic	**Benzoic acid** hinders glucose fermentation. pH-dependent enzymatic inhibition restricts yeast growth, inhibiting glycolysis and causing ATP depletion. Other organic acids share a similar mechanism as benzoic acid.	[102]
		At low pH, the uncharged and undissociated state freely diffuses across the plasma membrane into the cell.	[103]
		Increased turgor pressure and oxidative stress, depletion of ribosomal RNA, and other relevant cofactors.	[104]
*L. plantarum*	Fatty acids (**FA**) Short and long chain *3-Hydroxy Fatty Acids*	Cell membrane disruption; alteration of internal ultrastructure of the yeast cell. The shape and size of the cell wall remain unaltered.	[105]
		Partition of the lipid bilayers of cell membranes, resulting in (1) loss of membrane integrity, (2) increased membrane fluidity and permeability, uncontrollably releasing intracellular compounds.	[99]
		Detergent-like properties disrupt the cell membrane structure, increasing its permeability.	[106]
		Inhibition of ergosterol production, affecting the cell membrane, leading to cell death	[107]
*L. reuteri*	Reuterin	Reuterin's aldehyde group interacts with thiol groups of diverse molecules, causing oxidative stress.	[94]
*L. fermentum* *L. casei*	Biosurfactants	Exert anti-adhesive activity and induce detachment of already adherent *Candida* cells.	[93]

#### Bacteriocins and Bacteriocin-Like Substances

Bacteriocins are antimicrobial peptides, or peptide complexes, categorized into three major classes according to their synthesis and chemical structure [108, 109]. Bacteriocin-like substances (BLS) share traits similar to bacteriocins but do not entirely fit the typical criteria. Several reports suggest that putative lactobacilli-produced bacteriocins and BLS from cell culture supernatants exert anticandidal activity [110, 111]. However, only a handful of reports have studied the susceptibility of *Candida* to purified and biochemically characterized BLS and bacteriocins [86, 87, 112, 113]. The anticandidal mechanisms of action from lactobacilli-produced bacteriocins are practically unknown. The two-peptide plantaricin (a class IIb bacteriocin from *L. plantarum*) is the only purified bacteriocin whose effect on *C. albicans* physiology and morphology has been described. Plantaricin kills *C. albicans* by disrupting the cell membrane integrity due to depolarization and leakage of essential ions. Plantaricin also increases ROS production, inducing apoptosis [101]. The anticandidal activity of bacteriocins from bacteria other than lactobacilli –i.e., *Lactococcus (nisin), Streptococcus* (bacteriocin C3603), *Enterococcus* (bacteriocin Bac-GM17), among others- has also been studied by different groups [114–118].

#### Weak Organic Acids: Acetic, Lactic, Benzoic, and Sorbic Acids

Benzoic acid has antifungal activity caused by the disruption of glucose fermentation in yeast due to: (1) accumulation of hexose monophosphates; (2) decrease in intermediates beyond the phosphofructokinase cycle; and (3) pH-dependent enzymatic inhibition of phosphofructokinase and hexokinase, leading to subsequent inhibition of glycolysis that causes energy loss (ATP depletion). Other organic acids, such as sorbic acid and acetic acid, also inhibit the anaerobic fermentation on yeast and may share the same mechanism of action observed in benzoic acid [102]. The antifungal activity of benzoic acid is pH-dependent, as the undissociated acid (pKa = 4.2) is active only in acidic pH [119]. Low pH enables free diffusion of benzoic acid across the plasma membrane into the fungal cell [103], leading to intracellular acidification. This acidification causes turgor pressure increase, oxidative stress, and depletion of diverse molecules, including RNA [104]. Cottier et al. reported that acetic and butyric acids (and possibly other WOAs) reduce the *C. albicans* growth rate and change the expression of specific proteins like the putative glucose transporter Hgt16 protein and the predicted membrane transporter Orf19.7566, which mediate the fungistatic effects [120].

#### Fatty Acids

These acids disrupt the fungal membrane and the ultrastructural organization of the cell [105], resulting in growth inhibition of *C. albicans*. Specifically, fatty acids: (1) partition the lipid bilayers of cell membranes, resulting in loss of integrity and an increased fluidity and permeability, leading to an uncontrolled release of intracellular electrolytes and proteins [99]; (2) inhibit the production of ergosterol –an essential component of fungal cell membranes-, affecting the cell membrane integrity [107]; (3) compromise the structure of cell membranes due to their detergent-like properties [106]. Crowley et al. suggested that chain length may play an essential role in antifungal action, with long-chain fatty acids having the highest antifungal activity [99]; however, other studies show no apparent correlation between chain length and antifungal activity [105, 121].

#### Other Metabolites

##### Reuterin

Reuterin (3-hydroxypropionaldehyde acid) is a glycerol-derivative that displays fungicidal activity toward different *Candida* species [122]. The highly-reactive aldehyde group of reuterin may interact with thiol groups of small molecules and proteins, causing oxidative stress that results in growth inhibition [94]. Not surprisingly, reuterin increases the expression of OxyR, a transcriptional regulator that induces upregulation of oxidative stress genes.

##### Biosurfactants

Biosurfactants are compounds that contain both hydrophilic and hydrophobic moieties that reduce surface tension. Biosurfactants both hinder the ability of *C. albicans* to adhere to abiotic surfaces and tissues and display direct anticandidal activity. Although the modes of action of biosurfactants are still unknown, adherence reduction may be caused by changes in the cell wall charge, rendering the cell incapable of overcoming its electrostatic repulsion barrier with the substrate [123]. In addition to reducing adhesion, glycolipopeptide biosurfactants from *L. pentosus* exert direct anticandidal activity [124].

##### Enzymes

*L. johnsonii* produces enzymes with a chitinase-like activity, degrading the fungal cell wall of *C. glabrata via* enzymatic hydrolysis, leading to fungal growth inhibition [90].

#### Molecules From the Cell Surface

In addition to soluble metabolites, surface molecules from the cell wall have been identified to display anticandidal activity; however, their modes of action are still unknown. LGG cell wall-associated exopolysaccharides (EPS) reduce *C. albicans* hyphal transition and adhesion to epithelial cells [125].

### Indirect Antifungal Activity: *Lactobacillus*-Host-*Candida* Interaction-Based Mechanisms

*In vivo* studies in animal models –particularly in mice- show that lactobacilli can reduce *Candida* burdens and reverse dysbiosis. *Candida*-induced stomach lesions, gastric inflammation, and dysbiosis in murine models are attenuated by several species, i.e., *L. rhamnosus* [126], *L. gasseri, L. helveticus* [127], and *L. pentosus* [128], among others. Lactobacilli may also act as prophylactic agents. Immunosuppressed mice that ingested *L. rhamnosus* 14 days before inoculation with *Candida* showed a significant reduction in *C. albicans* numbers and inflammation [129]. Another *in vivo* study showed that *L. crispatus* and *L. fermentum* supernatants reduce *Candida* burdens in a vulvovaginal candidiasis murine model [130].

**Figure 2:**
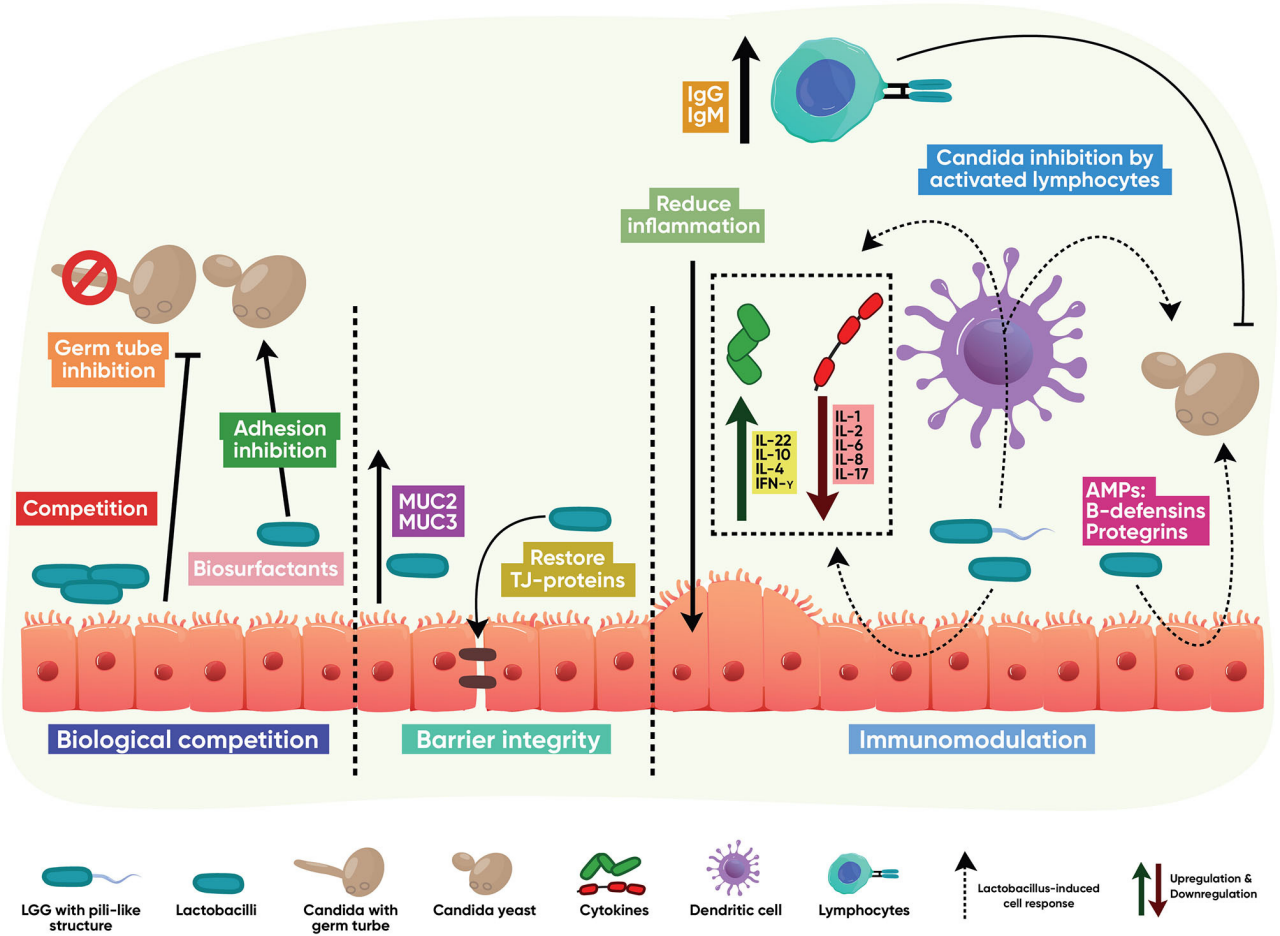
Indirect antifungal modes of action. *Lactobacillus* interacts with the host to stimulate protective, antifungal functions. These mainly result from competition with *Candida* in binding to mucus membrane glycoconjugates; strengthening the mucosal barrier through induction of mucins and positive regulation of tight junction proteins; activation of T and B cell immune responses; downregulation of host-destructive inflammation; and induction of innate epithelial antimicrobial peptides.

Lactobacilli-host-fungal interactions that control *Candida* infections mainly result from competition in binding to mucus membrane glycoconjugates; strengthening the mucosal barrier through induction of mucins and positive regulation of tight junction proteins; triggering a protective immune response; and biotransformation of host metabolites into active anticandidal compounds, as shown in Figure 2.

#### Biological Competition

Lactobacilli can reduce *Candida* colonization *via* competition, exclusion, and displacement mechanisms. Lactobacilli interact with GI tract mucus layer glycoconjugates to adhere avidly to the gut mucosa despite the constant changes in the intestinal environment [131]. *Lactobacillus* species adhere to epithelium resulting in reduced fungal adhesion and the ability to form mucosal biofilms [128, 132–135]. EPS from *L. rhamnosus* contribute to its adhesion to the host while reducing the epithelial adhesion of *C. albicans* [125]. Additionally, supernatants from *L. gasseri* and *L. crispatus* reduce the *C. albicans* ability to adhere to HeLa cells [136].

#### Immunomodulation

Lactobacilli display immunomodulatory activity by activating the immune response *via* classic surface-associated Microorganism-Associated Molecular Patterns (MAMPs). *Lactobacillus* MAMPS include the mannose-binding lectin Msa (*L. plantarum*), the cell-wall-associated hydrolases p40 and p75 (*L. rhamnosus*), and the S layer protein A –SlpA- (several *Lactobacillus* species). MAMPs induce a host response when interacting with host pattern recognition receptors [137]. The MAMP-induced host response can result in the prevention or reduction of *Candida* colonization, probably by their ability to activate Toll-like (TLRs) and NOD-like receptors (NLRs) [138]. The following epithelial antifungal effector mechanisms are reported to be influenced by lactobacilli:

##### Induction of Antimicrobial Peptides

Lactobacilli induce the production of several epithelial AMPs with anticandidal activity, such as β-defensins and protegrins [56, 139]. Vylkonova et al. showed that human β-defensins 2 and 3 disintegrate the cell wall of *C. albicans*, whereas only β-defensin 3 induces permeabilization on the cell membrane. Moreover, both β-defensins showed synergistic anticandidal activity with other AMPs –lysozyme and lactoferrin [140]. Rizzo et al. showed that β-defensin 2 and 3 upregulation is mediated by *L. crispatus* [139]. Protegrins are reported to disrupt the cell membrane of several microorganisms, including *Candida* species [141].

##### Anti-inflammatory Properties

Numerous *in vitro* reports confirm that different *Lactobacillus* species downregulate the expression of pro-apoptotic cytokines, i.e., TNFα- and several other pyroptotic or pro-inflammatory cytokines such as IL-1α, IL-1β, IL-2, IL-6, IL-8, IL-17, [56, 58, 89, 90, 139, 142]; whereas other studies showed that the anti-inflammatory cytokine IL-10 is upregulated [143, 144]. The pili-like structure from LGG reduces IL-6 expression while increasing the IL-10 production [54]. Tryptophan catabolism by lactobacilli has been reported to prevent colonization by *C. albicans*, as the production of indole derivatives from tryptophan degradation induces the production of IL-22 (an IL-10 group cytokine involved in mucosal barrier function and induction of epithelial AMPs) mediated by an aryl-hydrocarbon-receptor (AhR) [145]. Several *in vivo* studies –in murine models- confirm the modulatory effect of lactobacilli in cytokine production after *Candida* infection: *L. crispatus* and *L. delbrueckii* consumption increase IFN-γ, IL-4, and IgG expression while decreasing IL-17 expression [146], *L. rhamnosus* consumption reduces IL-6 production [129], and *L. reuteri* increases the expression IL-22 [147]. Authier et al. showed that combined oral administration of *L. helveticus* and *L. gasseri* reduced TNF-α, IL-1β, IL-8, and CRP inflammatory markers while increasing the expression of IL-1ra and IL-10 anti-inflammatory markers in colonic tissues of *C. albicans*-infected mice. Moreover, mRNA expression of enzymes involved in the synthesis of pro-inflammatory eicosanoids was also decreased [127]. Clinical trials also confirm the immunomodulatory properties of lactobacilli in humans. Macnaughtan et al. showed that *L. casei*-treated human patients with cirrhosis had lower plasma MIP-1B concentrations in an 8-week course; IL-1B and MPC-1 –in the alcoholic patient subgroup- and IL-17 –in the non-alcoholic cohort- plasma concentrations were also reduced [148]. Another clinical study reported that an *L. reuteri*-antibiotic combined therapy reduced abdominal pain and inflammatory markers (C-reactive protein levels) in acute uncomplicated diverticulitis patients; while also lowering hospitalization time [67]. The ability to reduce inflammation is critical in balancing immunopathology and protection in mucosal infections. For example, Swidsinski et al. showed that the colonic mucous layer is thinner in inflamed areas, allowing increased microbial adherence and infiltration [149].

##### Immune Cell Activation

*In vitro* analyses show that lactobacilli may activate immune cells by signaling *via*TLR4 and TLR9 [90, 150] binding to cell wall peptide SlpA [151] or cell wall peptidoglycans (PGN) [152]. *Lactobacillus acidophilus* promotes the migration of macrophages [89] and regulates T cell responses by interacting with dendritic cells [153]. The LGG pilus-like structure may modulate inflammatory response by signaling *via* TLR3 and TLR4 in intestinal epithelial cells [54]. Studies *in vivo* show that lactobacilli may induce a protective IgG and IgM- response [154] and regulate T cell responses [155]. Additionally, purified PGN from *L. rhamonosus* significantly increases the T-cell titer in malnourished mice challenged with pneumococcal infection [156]. Intravaginal inoculation of *L. crispatus* biosurfactants reduced the leukocyte influx in mice challenged with *C. albicans* [133]. A recent clinical study showed that HIV patients treated with *L. casei* displayed an increase in the CD4+/CD8+ ratio compared to the placebo group; yet, the difference did not reach statistical significance [157].

#### Mechanisms of Epithelial Barrier Protection

Several pathogens disrupt the mucosal barrier integrity *via* different mechanisms, such as compromising the production of mucins or the organization of tight junction proteins [158, 159]. *In vitro* assays show that lactobacilli improve the epithelial barrier function against pathogen invasion, contributing to protection of the GI tract from invasive pathogens [160]. Lactobacilli induce epithelial secretion of mucins MUC2 and MUC3, which improve barrier integrity by preventing microbial adhesion [143, 161, 162]. Zhou et al. suggested that the modulation of the mucosal homeostasis and MUC2 expression may be related to lactobacilli EPS [163]. Additionally, Yu et al. revealed that when the epithelium is challenged with pathogenic bacteria, *L. fructosus* preserved the intestinal epithelial cell integrity and the transepithelial electrical resistance, an intercellular tight junction integrity marker [164]. In a porcine intestinal epithelial cell line (IPEC-J2) *L. plantarum* ZLP001 pretreatment inhibits the reduction in tight junction proteins and the increase in gut permeability caused by enteropathogenic *E. coli* infection. Lactobacilli maintain barrier integrity by increasing the expression of tight junction proteins (claudin-1, occludin, and Zonula occludens-1), thereby protecting the GI tract from invasive pathogens [56]. Also, the ability of lactobacilli to downregulate inflammatory cytokine expression may indirectly preserve the integrity of tight junctions [165]. In organoid models, *L. reuteri* can stimulate the proliferation of intestinal epithelia by increasing expression of R-spondins, leading to activation of the Wnt/β-catenin pathway [166].

*In vivo* studies in murine models also confirm the role of lactobacilli in preserving the integrity of the mucosal barrier. Recent studies showed that desmosome-like junctions –in vaginal epithelium- are reduced during *Candida* infections; however, ultrastructural analysis revealed that *L. crispatus* and *L. delbrueckii* increase the number of desmosome-like junctions to almost the numbers in uninfected epithelia [146]. Oral administration of *L. reuteri* reduced the colonization and intestinal inflammation in mice infected with the mucosal pathogen *Citrobacter rodentium* [166]. In induced epithelial barrier hyper-permeability mice, *L. rhamnosus* restored the expression of apical junction proteins Claudin-4, F11r, E-cadherin and occludin, confirming the role of lactobacilli in the modulation of junction proteins [167]. Moreover, *L. reuteri* ameliorated intestinal mucosa damage in DSS-treated mice by increasing the intestinal stem cell marker Lgr5^+^ and the antibacterial enzyme lysozyme [147].

#### Biotransformation of Host Compounds

Lactobacilli may metabolize host-produced macromolecules producing secondary metabolites with antimicrobial activity. This mechanism has been explored for bacteria but remains unexplored for *Candida*. Recently, McNair et al. reported a new casein-derived *Lactobacillus*-biotransformed peptide with antifungal activity [168].

## Conclusions and Future Directions

Current evidence regarding the anticandidal activities of *Lactobacillus* supports their use as a promising therapeutic alternative agent for preventing and treating candidiasis; yet, several challenges exist in establishing how lactobacilli inhibit *Candida* growth. The production and/or activity of antifungal compounds *in vitro* can be affected by the type of culture media, pH, incubation time, solid vs. liquid culture, and the interaction of lactobacilli with other microorganisms which are part of the same ecosystem [98, 169–172]. Culture conditions influence both the qualitative composition and quantitative aspects of anticandidal metabolites; therefore, diverse metabolite profiles and activity may arise under non-standardized experiments among different laboratories [100]. For example, the composition of culture media influences bacteriocin production in *L. pentosus* [173]. Another example is the variable anticandidal activity of *L. johnsonii* in liquid vs. solid media. The anticandidal properties of *L. johnsonii* are usually reported as “good activity” in broth; in contrast, when the susceptibility assays are performed on agar plates, the inhibition is reported as “weak” or “null” [174–178]. Variable experimental conditions may also influence the detection sensitivity of specific metabolites, leading to contradictory results and potentially erroneous data interpretation. Also, lack of standardization negatively influences assay reproducibility among different laboratories. Many of these compounds are rarely examined under controlled pH conditions [98], which is critical for better understanding their activity. A physiologic range of pH should be maintained during antifungal activity assays to reflect the dynamic changes of the host tissue environment in health and disease. The interaction with other microorganisms also plays a role in lactobacilli metabolism. For example, certain strains of *L. plantarum* produced bacteriocins only when co-cultured with other microbial species in liquid media *in vitro* [170].

By regulating the growth of other microorganisms lactobacilli may maintain a healthy microbiome and inhibit dysbiotic disease states. Nevertheless, more research regarding the mutual effect of *Lactobacillus* spp. with other probiotics and other microorganisms –commensal or pathogenic- is needed. The presence, composition, and metabolic stage of the resident microbiota may affect the efficacy of *Lactobacillus* species used as probiotics. For example, while lactobacilli modulate the growth and morphologic transition of *Candida in vitro*, murine studies show that *Candida* overgrowth may reduce the abundance of oral lactobacilli, favoring an enterococcus-rich dysbiosis, as recently demonstrated by our group [17, 23]. Moreover, *Candida* may prevent the restoration of the healthy microbiota post-antibiotics, by inhibiting *Lactobacillus* spp in th murine gut [40].

Beyond the influence of lactobacilli on the host immune system, the indirect anticandidal mechanisms of lactobacilli remain relatively underexplored. Several connections between lactobacilli and host metabolism have been established; however, a comprehensive network of the complex metabolic interactions between probiotic lactobacilli and *C. albicans* remains to be characterized both *in vitro* and *in vivo*.

Lactobacilli-produced metabolites (post-biotics) also remain underexplored. Understanding the mechanism of action behind the anticandidal compounds is critical for assessing implications to non-pathogenic host microbiota, potential adverse effects, and their prospective therapeutically use as auxiliary anticandidal agents. The interactions between lactobacilli-produced antimicrobial post-biotics and current conventional therapies –including antibiotics- remain practically unknown. Would these anticandidal post-biotics display synergistic activity with current antifungal treatments?

In conclusion, lactobacilli exert direct and indirect activity against *Candida* species *via* a wide variety of mechanisms. As these organisms are among the most consumed probiotics, understanding their mechanisms of action is clinically relevant. While the pace of new anticandidal drug discovery remains slow and drug-resistance is becoming a serious threat to humans and animals worldwide, *Lactobacillus-*based antifungals may be used as effective adjunctive therapies in several mucosal fungal diseases.

## Author Contributions

All authors contributed in the design and the preparation of the manuscript and have approved the final version of the manuscript.

## Conflict of Interest

The authors declare that the research was conducted in the absence of any commercial or financial relationships that could be construed as a potential conflict of interest.

## Glossary

**Commensal:** Refers to organisms that benefit from living within the host, while the host experiences no net effect. However, our understanding of microbial commensalism is limited. Also, commensal interactions may change under different host conditions [179–181].

**Paraprobiotics:** (Also known as “non-viable” or “inactivated” probiotics) refer to non-viable microbial cells that confer health benefits to the host. Paraprobiotics are usually consumed in diverse “healthy” food products or supplements [57].

**Pathobionts:** Commensal microorganisms that can become opportunistic pathogens associated with chronic inflammatory conditions. Pathobionts are not pathogenic to the host under normal conditions [182].

**Pathogen:** Is a microorganism that causes disease to its host. They are also called infectious agents, as they may cause infections [183].

**Post-biotics:** (Metabiotics, biogenics, or metabolites/CFS (cell-free supernatants) are soluble factors (metabolites) secreted by live bacteria or released after bacterial lysis. The bioactivity of these post-biotics offers health benefits to the host [57].

**Probiotics:** Live microorganisms that confer health benefits to the host. Probiotics are usually consumed in cultured milk and fermented foods.

**Symbiosis:** In the host-microbiota context, symbiosis encompasses different interactions, from commensalism to mutualistic (win-win) [184, 185].
